# Generalized Anxiety Disorder in Very Young Children: First Case Reports on Stability and Developmental Considerations

**DOI:** 10.1155/2018/7093178

**Published:** 2018-09-24

**Authors:** Michael S. Scheeringa, Lauren C. Burns

**Affiliations:** Department of Psychiatry and Behavioral Sciences, Tulane University School of Medicine, 1430 Tulane Ave., #8448, New Orleans, LA 70112, USA

## Abstract

Generalized anxiety disorder (GAD) is purported to start in early childhood but concerns about attenuation of anxiety symptoms over time and the development of emerging cognitive and emotional processing capabilities pose multiple challenges for accurate detection. This paper presents the first known case reports of very young children with GAD to examine these developmental challenges at the item level. Three children, five-to-six years of age, were assessed with the Diagnostic Infant and Preschool Assessment twice in a test-retest reliability study. One case appeared to show attenuation of the worries during the test-retest period based on caregiver report but not when followed over two years. The other two cases showed stability of the full complement of diagnostic criteria. The cases were useful for demonstrating that the current diagnostic criteria appear adequate for this developmental period. The challenges of accurate assessment of young children that might cause missed diagnoses are discussed. Future research on the underlying dysregulation of negative emotionality and long-term follow-ups are needed to better understand the etiology, treatment, and course of GAD in this age group.

## 1. Introduction

In the fifth edition of the Diagnostic and Statistical Manual (DSM-5) [1], generalized anxiety disorder (GAD) is described in a seemingly contradictory fashion in the sense that most adults who are diagnosed report that they have felt anxious all of their lives but the onset of the disorder is rare prior to adolescence. This incongruity suggests either that GAD onset in early childhood is rare and the anxiety symptoms manifested by individuals prior to adolescence will evolve later into GAD, or that GAD onset in early childhood is common and detection of GAD during this age is complicated by developmental challenges. Chief among these developmental challenges may be the stability of symptom manifestations. In Egger et al.'s (2006) survey of two-to-five-year-old children, test-retest reliability kappa for GAD when assessed one week apart was 0.39, which was the second lowest among 12 disorders assessed [2]. They found significant attenuation of the diagnosis by the second interview for six disorders, and the largest effect for attenuation was found for GAD (Odds Ratio = 1.8), but no details were provided about which criterion may have contributed to this low stability of the diagnosis.

Other developmental challenges include that the types of worries required in criterion A (i.e., excessive worry about a number of things) may be different compared to older youths and adults. Young children may be more likely to seek excessive reassurance than to ruminate about other types of worries [3]. Their young age and dependence on caregivers may make their worries indistinguishable from separation anxiety disorder [4]. Young children do not attend work or school, so their worries may necessarily focus more on family and peers. In addition, the number of worries required in criterion A may be fewer compared to adults. Young children live within smaller social contexts, so they may have a fewer number of worries compared to older populations [5, 6]. To date, these have only been speculations and have not been supported with data from actual diagnosable cases in very young children.

Whether to include uncontrollability as a criterion is also relevant because, in contrast to the DSM-5, the International Classification of Disease definition does not require uncontrollability. The requirement of uncontrollability in criterion B is highly internalized and may be difficult to detect by caregivers. The capacities for self-reflection and meta-cognition are just emerging at seven years of age [7]. These emerging capacities may also make it challenging to detect the concomitant physiological symptoms of anxiety required in criterion C.

Despite the developmental challenges, recent research suggests that GAD does exist as a distinct and differentiated form in very young children. In a random population sample of 1,110 two- and three-year-old children, researchers used confirmatory factor analysis to test whether anxiety symptoms aggregated in a manner consistent with the taxonomy of GAD, obsessive compulsive disorder, separation anxiety disorder, and social phobia [8]. The data significantly fit the model of these four disorders; however a limitation was that the measure of anxiety symptoms did not include a comprehensive list of all GAD items.

Identifying GAD at an early age may be important because it has been shown for some psychiatric disorders that childhood onset portends a worse prognosis compared to adult onset. For example, childhood onset of conduct disorder [9] or schizophrenia [10] predicts more persistent and more impairing problems compared to adolescent or adult onset of those disorders. In a retrospective analysis of adult patients with GAD, researchers empirically demonstrated a bimodal age of onset at 24 years, but they did not examine earlier possible onsets in childhood [11]. In one of the few studies to examine differences in GAD based on age cohorts in childhood and adolescence, researchers found few differences on GAD symptom patterns in seven-to-nine-year-old children compared to ten-to-fourteen-year-old children [12]. However, the researchers did not ask about the age of onset of the symptoms for the two groups.

Identifying GAD at an early age may also be important for treatment advances because there may be different underlying or associated factors of the GAD phenotype at different ages. For example, recent research has suggested that anxiety sensitivity may be an important predictor of symptom chronicity. Anxiety sensitivity refers to the fear of experiencing anxiety and the belief that experiencing anxiety will lead to harmful social and physical consequences. In a prospective follow-up of 277 adolescents, those with elevated anxiety sensitivity were more likely to have high and increasing GAD symptoms over time [13]. Furthermore, in a meta-analysis of nine domains of emotional competence, significant moderating effects of age were found in two of the domains [14]. Younger children had more difficulty recognizing others' emotions whereas older children tended to use more externalizing coping strategies. These age differences may be potential targets for treatment advances in young children accurately identified to suffer from GAD.

There are only two known case reports of children six years and younger with GAD symptoms. A two-year-old child expressed worries about dirt, damage to her toys, and the fact that she would get hurt in the bathtub. These appeared uncontrollable as she could not forget about them enough to have fun in other activities. She manifested the criteria of concomitant physiological symptoms with irritability and restlessness [15]. A six-year-old boy woke several times during the night ruminating about whether he had injured a classmate the day prior [16]. Other worries included potential harm to himself or animals and that a favorite toy might melt in the car. He had clear elements of uncontrollability and concomitant physiological symptoms of fatigue and restlessness. Neither of these cases included formal examinations of the stability of symptoms.

Two treatment studies have been conducted with very young anxious children but GAD was mixed with other anxiety disorders [4, 17]. One assessment study has been conducted with two- to five-year-old children to describe differences of children with GAD compared to selective mutism, but test-retest stability of diagnoses was not tested and details at the item level of GAD were not described [18]. The aim of this paper was to present the first series of cases of children six years and younger with potential GAD with a comprehensive diagnostic instrument that was administered twice in a test-retest reliability study to examine the short-term stability in detail at the criterion level.

## 2. Method

The children were participants in a test-retest study for the Diagnostic Infant Preschool Assessment (DIPA). Children were recruited as consecutive intakes from an outpatient clinic. The interviewers were research assistants who received extensive training and ongoing supervision. Prior to their first interviews, they received formal training in the administration of the DIPA from the developer of the instrument and watched three videos by other interviewers. All of their interviews were videotaped and reviewed with the first author in order to maintain accurate understanding of symptoms and to maintain fidelity of technique.

The DIPA is an interview of caregivers about their children from late in the first year of life through six years [19]. It includes all symptoms for 13 DSM-5 disorders, but only the modules for posttraumatic stress disorder (PTSD), disruptive mood dysregulation disorder (DMDD), attention-deficit/hyperactivity disorder (ADHD), oppositional defiant disorder (ODD), separation anxiety disorder (SAD), and GAD were used in this study.

Each symptom question begins with a stem question, which the interviewer reads verbatim. After a stem question, the interviewer uses his/her judgment on the number of follow-up probes needed. DIPA questions are worded explicitly to ask about symptoms by framing behaviors as “problem” behaviors, “excessive,” “often,” “too much,” or things that children “have trouble with.” Caregivers are often asked if their children show a certain behavior* “more than the average child his/her age,”* which is an important frame of reference given the developmental differences both within and beyond the preschool period. A simple yes or no response from a respondent is never accepted as sufficient, as interviewers are instructed to get an example of every symptom to verify (or disprove) respondents' answers.

The DIPA assesses functional impairment in a disorder-specific fashion by asking about impairment at the end of each disorder. Five areas of role functioning, with parents, with siblings, with peers, at school/day care, and in public, were assessed. Impairment was counted if at least one of those areas was endorsed.

For each area of role functioning except school/day care an additional question is asked if caregivers make accommodations so that children do not manifest their impairment. For instance, if a caregiver answers that her child does not have problems when taken out in public but answers that she accommodates by almost never taking her child out in public, this is counted as functional impairment.

The protocol was approved by the Tulane University Committee on the Use of Human Subjects. Clinicians who conducted the clinical intakes asked caregivers at their initial meetings if research assistants could approach them about the study. If they agreed, research assistants obtained written consent from the caregivers, and then collection of data proceeded with research assistants in a private office. At the conclusion of the first interview, they were scheduled to come back one-to-two weeks later for the second interview with a different research assistant. Identical measures were collected at the first and second interviews. The results of the DIPA were shared with the treating clinicians. The names of the children have been changed and all personally identifying information has been omitted from case reports.

## 3. Case Presentations

*Case 1*. Dustin, a five-year-old White male, was brought to the clinic by his mother for her chief concern of defiance and emotional dysregulation. During the first diagnostic interview, Dustin's worries included any social situations that included scrutiny from others (e.g., he refused to stand in front of the church for his baptism), death, feeling anxious in places where he does not have control, and intense fear of bugs. Worries occurred almost daily and appeared somewhat uncontrollable. At the second interview, two weeks later, his worries remained the same but now included getting lost. At school, his negative emotionality could escalate into tantrums of screaming and trying to bang his head on a wall, which could last two hours. At home, getting him to take a bath could involve an hour of crying and protesting. Symptoms first appeared at one-and-a-half years but because he was preverbal at that age his mother could not give examples that clearly met GAD criteria.

Physical symptoms present during Dustin's periods of worry included feeling restless, on edge, difficulty concentrating, and irritability. Functional impairment included a slight impact on parental relationships, a moderate impact on the relationship with his daycare provider, and a severe impact on the child's ability to go out in public. His mother almost always accommodated him by rarely taking him outside of the home.

Dustin met all of the criteria for GAD, ODD, and SAD. His treatment involved helping learn ways to calm himself and help his parents manage his behavior. He improved markedly by the end of the school year and treatment terminated over the summer. But his behavior flared up when school resumed and treatment had to be restarted.

*Case 2. *Ivan, a five-year-old white male, was brought to the clinic by his mother for her chief concern of his worries about death. During the first diagnostic interview, Ivan was described as having excessive worries related to something happening to his family, death, Earth being sucked into a black hole, criminals harming someone in the family, and separating from parents when going to school. He worried that other children did not like him and that he would not do a good job on tasks. Symptoms first appeared at age four years.

His worries appeared clearly uncontrollable to his mother. Physical symptoms present during Ivan's periods of worry included difficulty concentrating. Functional impairment was endorsed in the parental relationship.

No excessive worries were endorsed during the second diagnostic interview even though those interviews were only two weeks apart. However, when his medical record was reviewed for this paper, it was clear that he had the same worries consistently for two years following those interviews up to the present time.

Ivan met all of the criteria for GAD plus SAD. He had not yet improved markedly after two years of treatment.

*Case 3. *Alani was a six-year-old Pacific Islander female who was brought to the clinic by her mother for her chief concern of fear of bad weather. No other excessive worries were endorsed during the first interview, so her fear of weather was conceptualized as PTSD initially. During the second interview however Alani was described as having excessive worries related to peers making fun of her, grades, sickness, and worries about the safety of other people. Her treating clinician had been unaware of these. Her worries appeared clearly uncontrollable to her mother. Her symptoms first appeared at age four years.

Physical symptoms present during Alani's worry episodes included restlessness, feeling on edge, irritability, and problems sleeping. No functional impairments or accommodations were endorsed by her mother. Alani's clinician reported however that Alani experienced marked impairment due to her worries and severe restrictions on her activities, as her impairment was a central focus of the therapy.

Alani met all of the criteria for GAD, PTSD, and ODD. She improved markedly over thirteen sessions of psychotherapy.

## 4. Discussion

This paper presents the first known data on short-term stability of diagnostic criteria of GAD in help-seeking cases of very young children. Consistent with Egger and colleagues (2006), attenuation of the diagnosis appeared to occur for Ivan because his mother did not endorse any worries at the second interview. When followed for two more years during treatment however it was clear that his worries had not disappeared in the least. This suggests that so-called attenuation may be due to interviewing technique by investigators rather than disappearance of the disorder. Alternatively, the appearance of attenuation would be consistent with Weems' model (2008) which attempts to explain the inconsistencies on the stability of childhood anxiety disorders as a core underlying dysregulated anxiety response system and views disorder-specific symptoms (such as GAD) as secondary characteristics that may be triggered by social and environmental contexts at different points in development [20]. This model suggests that if the social and environmental contexts changed for Ivan, his symptoms may flare or relapse accordingly consistent with an underlying temperament of negative emotionality that manifests as GAD under stress and the symptoms diminish when the stress has abated.

In this first-ever item-level analysis of the GAD criteria in young children, the DSM-5 diagnostic criteria do not appear to need developmental modifications to accommodate young children. These data contradict speculations in the literature that the types of worries [3] and the number of worries [5, 6] may be different compared to older age groups. The A criterion that excessive worry occurred more days than not for at least six months was easily met in all of our cases. The additional requirement that this symptom be manifest about a number of events or activities was also met. In our cases, the minimum number of types of worries was three and in the other cases parents could easily identify five types of worries. There was a wide range of the types of worries, and there was no support to try to narrow them down into a few categories (e.g., family and peers) [3, 4].

The B criterion is that the person finds it difficult to control the worries. It was anticipated that this would be problematic to detect if young children had to verbalize an internalized state of feeling out of control. Contrary to expectations, uncontrollability was apparent in all of the cases.

The diagnostic criterion of uncontrollability appears to have elements in common with anxiety sensitivity with the belief that experiencing anxiety will snowball into additional harmful social and physical consequences. Because elevated anxiety sensitivity has been associated with high and increasing GAD symptoms over time [13], treatment approaches that target uncontrollability and address anxiety sensitivity would be supported in this age group. The C criterion is that the worries are associated with physiological symptoms such as restlessness, difficulty concentrating, or irritability. Three out of six possible items are required in adults but only one out of six is required for children even though there is no known empirical evidence to support the threshold of one item in children. The three cases showed one, four, and four criterion C symptoms. The child with only one physiological symptom was Ivan, which was also the case in which his mother did not endorse worries at the second interview. If Ivan's mother underestimated the extent of his worries at the second interview, it is conceivable that she also underestimated the extent of his physiological symptoms at the first interview. Nevertheless, it does not appear that the number of physiological symptoms that are required needs modification.

The D criterion is that the symptoms cause clinically significant distress or impairment. It has been suggested that impairment not be required in this age group because the excessive worries are by definition not developmentally normative (suggesting that impairment is unnecessary) [21]. This criterion was met however in all three cases.

While the DSM-5 diagnostic criteria do not appear to need developmental modifications to accommodate young children, the key for accurate diagnosis appears to be assessment technique. One of the GAD diagnoses was not recognized by the treating clinician and was only diagnosed after the caregiver was interviewed with the structured and comprehensive DIPA instrument. Assessment of anxiety disorders in young children is challenging but vitally important. Delayed diagnosis may unnecessarily prolong avoidable suffering regardless of the etiological mechanisms [15]. Furthermore, because age of onset may be an important factor for prognosis or treatment response, early detection may spur much-needed research in this age group.

Extension of recent work on underlying emotional processes on adolescents with anxiety disorders is needed for younger children. Potential avenues include treatment approaches that target anxiety sensitivity [13] and the greater difficulty that younger children have with recognizing others' emotions [14]. Future research on the underlying dysregulation of negative emotionality [20] and prospective long-term follow-up of young children are likely to be productive areas to understand how to help children during this time of rapid developmental changes.
